# Enzymatic Hydrolysis-Assisted Separation and Purification of High F-Value Oligopeptides from Sea Cucumbers and Their Anti-Fatigue Mechanism

**DOI:** 10.3390/md24010010

**Published:** 2025-12-23

**Authors:** Xin Mu, Xinxin Yang, Jian Jiao, Ming Du, Zhenyu Wang

**Affiliations:** 1 SKL of Marine Food Processing & Safety Control, School of Food Science and Technology, Dalian Polytechnic University, Dalian 116034, China; gutou0506@163.com (X.M.); 15841614642@163.com (X.Y.); duming@dlpu.edu.cn (M.D.); 2 National Engineering Research Center of Seafood, Collaborative Innovation Centre of Seafood Deep Processing, Dalian Polytechnic University, Dalian 116034, China; jiaojian@trtjkdl.com; 3 Beijing Tong Ren Tang Health (Dalian) Marine Food Co., Ltd., Dalian 116041, China

**Keywords:** two-step enzymatic hydrolysis, simultaneous separation of polysaccharides and peptides, de-aromatization, free radical, metabolic byproducts

## Abstract

Sea cucumber peptides have been shown to possess a number of functions, including antioxidant, anti-inflammatory, anti-tumor, and anti-fatigue effects, as well as immune regulation and promotion of collagen synthesis. Among these, high F-value oligopeptides are a promising natural active ingredient demonstrating excellent anti-fatigue effects. This study utilized fresh sea cucumbers as the primary raw material, employing membrane separation technology to investigate the simultaneous separation of sea cucumber polysaccharides and peptides. The process for removing aromatic amino acids during the preparation of high F-value oligopeptides from sea cucumbers was optimized, and the mechanism underlying their anti-fatigue effects was explored. A two-step enzymatic hydrolysis method using neutral protease and composite flavor protease was employed, followed by membrane separation using a 10,000 Da molecular weight ultrafiltration membrane, yielding a sea cucumber peptide yield of 45.00 ± 0.12% and a sea cucumber polysaccharide yield of 51.28 ± 0.63%. Following the removal of aromatic amino acids by means of activated carbon adsorption, the F-value of the high-F-value oligopeptides attained 23.82, with a yield of 24.56%. The experimental findings demonstrated that high-F-value oligopeptides exhibited a substantial increase in the swimming duration of mice and a notable enhancement in their grip strength. These observations signified their substantial anti-fatigue potential. Furthermore, studies have indicated that sea cucumber high-F-value oligopeptides reduce metabolites produced by exercise, enhance muscle protection, increase the activity of antioxidant enzymes in the body, and alleviate fatigue, thereby achieving an anti-fatigue effect.

## 1. Introduction

Sea cucumbers are highly nutritious, being rich in various nutrients, and are a high-protein, low-fat food that is beneficial to human health [1,2]. The body wall of the sea cucumber, which is the edible portion, is rich in proteins, primarily collagen, and sulfated polysaccharides [3]. This component accounts for between 60% and 80% of the dry weight [4]. The amino acid composition of these collagen proteins is scientifically balanced, facilitating facile absorption and utilization by the human body. The process of obtaining sea cucumber peptides involves a series of steps, including protease hydrolysis and separation purification. These peptides are characterized by their small molecular weight, ease of absorption, and high nutritional value. A plethora of studies have demonstrated that sea cucumber peptides possess a variety of biological activities, including antioxidant, antihypertensive, hypoglycaemic, anti-fatigue, antineoplastic, antibacterial, immune system-enhancing, and memory-improving effects [5,6].

The advanced processing of sea cucumbers is chiefly concerned with the extraction and utilization of bioactive substances, including sea cucumber peptides and polysaccharides. These substances can be formulated into a variety of products, such as capsules, powders and oral liquids. In the production process, enzymatic hydrolysis technology is employed for the extraction of active components, such as sea cucumber peptides [7]. These peptides are subsequently amalgamated with traditional Chinese medicines, which are both food and medicine, with a view to enhancing the overall health benefits of the product. The growing demand for health and wellness among consumers has led to significant market potential for sea cucumber health supplements, attracting the attention of consumers of all ages.

High F-value oligopeptides (HFO) refer to short-chain polymers composed of amino acids, and the F-value denotes the molar ratio of branched-chain amino acids to aromatic amino acids in a peptide mixture. In conditions of elevated F-value, oligopeptides demonstrate remarkable biological activity, particularly in domains such as the alleviation or treatment of liver disease [8,9], the relief of phenylketonuria [10,11], the regulation of immune function, the promotion of cellular repair, the antioxidant effects, and the anti-inflammatory properties. Therefore, oligopeptides with high F-value are widely considered to have better biological effects in alleviating fatigue [12].

It has been established that fatigue is closely associated with disease. Research hypothesizes that chronic fatigue may serve as a precursor to various health issues, including but not limited to cardiovascular disease, diabetes, depression, and chronic fatigue syndrome. Fatigue has been demonstrated to impair immune system function and induce inflammatory responses within the body, thereby weakening the body’s ability to adapt to fatigue and increasing an individual’s susceptibility to infection [13]. Furthermore, research confirms that fatigue may trigger mental health issues, creating a vicious cycle [14,15]. As societal pace accelerates and work pressures intensify, fatigue has become an increasingly prominent issue, drawing significant research attention in recent years.

The physiological basis of fatigue comprises its effects on the neuroendocrine system, immune system and metabolic functions. The theory of internal environmental instability highlights the disruption of homeostasis in physiological systems in the presence of fatigue [16]. The energy depletion theory posits that fatigue primarily stems from the depletion of energy reserves within the body, leading to impaired bodily functions and the sensation of fatigue [17,18]. The free radical theory of fatigue is predicated on the premise that oxidative stress and free radicals play a pivotal role in the process of fatigue production [19,20]. The metabolic byproduct accumulation theory posits that increased exercise intensity leads to the accumulation of acidic substances, such as hydrogen ions and lactic acid, in muscles and blood [21]. The mechanism by which high-F-value oligopeptides alleviate fatigue is one of the key issues that must be addressed in developing active products containing these compounds.

Despite the documented evidence that sea cucumber polysaccharides and peptides possess bioactivities that are beneficial to humans, there is currently no established technology for their simultaneous separation. The development of an efficient method for the simultaneous separation of polysaccharides and peptides from sea cucumbers is of fundamental importance. Such a method would enable the extraction of high-purity bioactive components, which in turn forms the basis for subsequent biological research and applications. In addition, while it is now evident that HFO possess anti-fatigue activity, no research has yet been conducted on the underlying anti-fatigue mechanism of HFO from sea cucumber body wall protein. This study utilizes sea cucumber body wall protein as the primary raw material, employing proteolytic hydrolysis and de-aromatization processes to achieve simultaneous separation of sea cucumber polysaccharides and peptides, in addition to the preparation of HFO. Then, a series of experiments were conducted using mice to investigate the anti-fatigue mechanism of HFO derived from sea cucumbers. This has considerable implications for the training, rehabilitation, and health management of the general population, as well as for athletes. The research findings will provide significant support for the development and utilization of marine biological resources, the development of health products, and further scientific research.

## 2. Results and Discussion

### 2.1. Improvement of Two-Stage Enzymatic Hydrolysis Conditions

**Figure 1:**
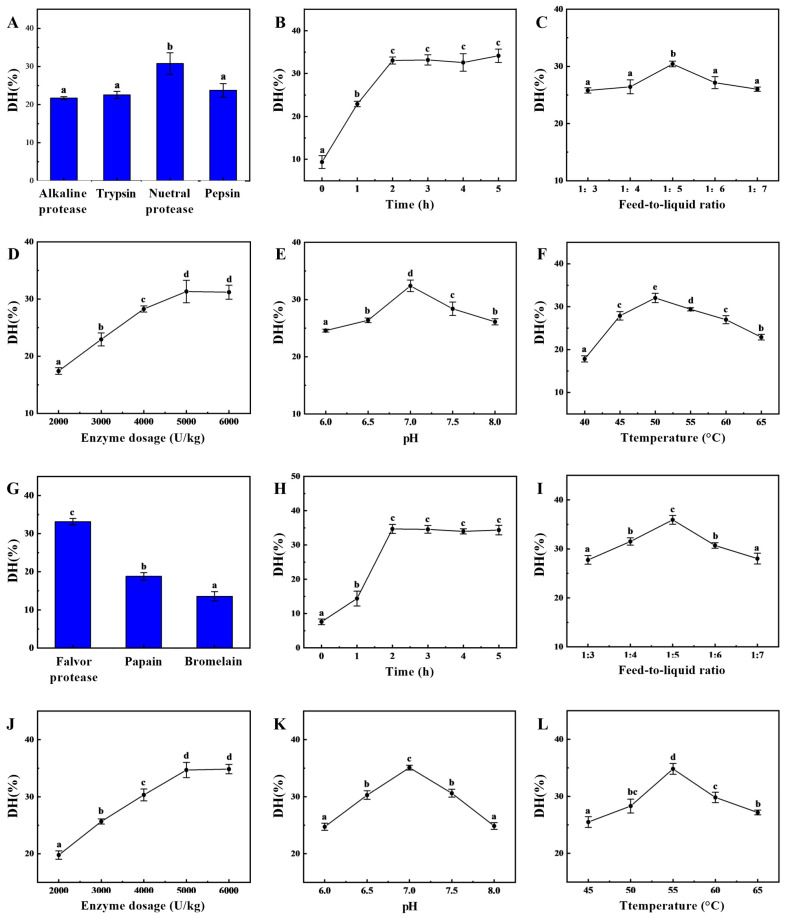
Improvements of two-step enzymatic hydrolysis process conditions. The degree of hydrolysis (DH) of sea cucumber body wall proteins is influenced by enzyme (**A**), neutropin hydrolysis time (**B**), feed-to-liquid ratio (**C**), enzyme dosage (**D**), pH (**E**), and temperature (**F**). Moreover, the degree of hydrolysis of sea cucumber body wall proteins can be further enhanced during the two-stage enzymatic hydrolysis process due to the influence of enzyme (**G**), flavor protease hydrolysis time (**H**), feed-to-liquid ratio (**I**), enzyme dosage (**J**), pH (**K**), and temperature (**L**). (Note: Lowercase letters in the figure indicate significant differences (*p* < 0.05) in the molecular weight of enzymatic hydrolysates from different enzymes).

In the first stage of enzymatic hydrolysis, the type of enzyme significantly affects the degree of hydrolysis, as shown in Figure 1A. The neutral protease produced significantly higher degree of hydrolysis than the other three endopeptidases, indicating that neutral protease had the best enzymatic hydrolysis effect, with a degree of hydrolysis of 30.78 ± 2.82%, which was similar to our previous experimental findings [22]. During this initial stage of enzymatic hydrolysis, the substrate concentration was high, resulting in a fast hydrolysis rate and the gradual accumulation of hydrolysis products. As time progressed, the substrate concentration decreased, leading to a slower hydrolysis rate. The hydrolysis degree increased gradually before two hours (Figure 1B), reached a peak at two hours, and then stabilized with no significant differences afterwards. The feed-to-liquid ratio directly affects enzyme activity and reaction efficiency (Figure 1C). At an appropriate feed-to-liquid ratio, both effective substrate utilization and high enzyme activity can be maintained to promote the reaction. Between a ratio of 1:3 and 1:5, the degree of hydrolysis gradually increases. However, as the feed-to-liquid ratio increases, the degree of hydrolysis shows a decreasing trend. At higher substrate concentrations, enzyme site saturation may lead to an increase in reaction rate, but it may also enhance enzyme inhibition. The effects of different enzyme dosages on sea cucumber hydrolysis are compared (Figure 1D). As the enzyme dosage increases, the degree of hydrolysis also gradually increases, with significant differences observed. The maximum hydrolysis degree is reached at an enzyme dosage of 5000 U/kg (31.32 ± 1.97%), after which it plateaus at dosages of 5000 U/kg and 6000 U/kg. Typically, neutral proteases exhibit optimal enzyme activity within a pH range of 6.0–8.0. If the pH is too low (acidic) or too high (alkaline), the enzyme conformation may change, causing the active site to lose function and thereby reducing enzymatic efficiency. Therefore, setting the pH to 7.0 can significantly enhance the degree of protein hydrolysis and improve the efficiency of the enzymatic process (Figure 1E). An appropriate temperature can increase the kinetic energy of enzyme molecules, promoting their binding with substrates and enhancing the rate of enzymatic hydrolysis (Figure 1F). However, when the temperature exceeds 65 °C, the enzyme structure may denature, leading to reduced activity or even complete inactivation. As the temperature increases from 40 °C to 50 °C, the hydrolysis rate first accelerates and then slows down. The extent to which the hydrolysis equilibrium shifts towards the forward direction is greatest at 50 °C, achieving a maximum hydrolysis degree of 32.04 ± 1.09%. Based on the above improvements, the enzymatic hydrolysis conditions for the initial stage can be determined as follows: a feed-to-liquid ratio of 1:5, an enzyme dosage of 5000 U/kg, a temperature of 50 °C, and a pH of 7.0, with hydrolysis conducted for 2 h.

Generally, a single protease exhibits selectivity toward hydrolyzing peptide bonds. To further enhance hydrolysis efficiency, secondary enzymatic hydrolysis can be employed to shift the hydrolysis site, thereby significantly improving enzymatic hydrolysis efficiency [7]. Figure 1G–L show that the degree of hydrolysis in the composite flavor protease group was significantly higher than that in the other groups, indicating that it had the greatest capacity for degrading protein substrates, with a degree of hydrolysis of 33.18 ± 0.83%. Based on the results of the single-factor experiment, the optimal conditions for the two-stage enzymatic hydrolysis were determined to be a hydrolysis time of 3 h, a solid-to-liquid ratio of 1:5, an enzyme dosage of 5000 U/kg, a pH of 7.0 and a hydrolysis temperature of 55 °C. After neutral protease hydrolysis for two hours, composite flavor protease was added, and hydrolysis continued for three hours. The hydrolysis degree then stopped increasing and reached a maximum value of 54.60 ± 1.55%, over 20% higher than the maximum achieved by single-enzyme hydrolysis. The molecular weight distribution of the enzymatic hydrolysate is shown in Supplementary Figure S1, with a significant increase in the proportion of components below 1000 Da. This facilitates both the separation of oligopeptides from macromolecular polysaccharides and the efficient absorption and physiological function of the oligopeptides.

### 2.2. Separation of Sea Cucumber Polysaccharides and Peptides

**Figure 2:**
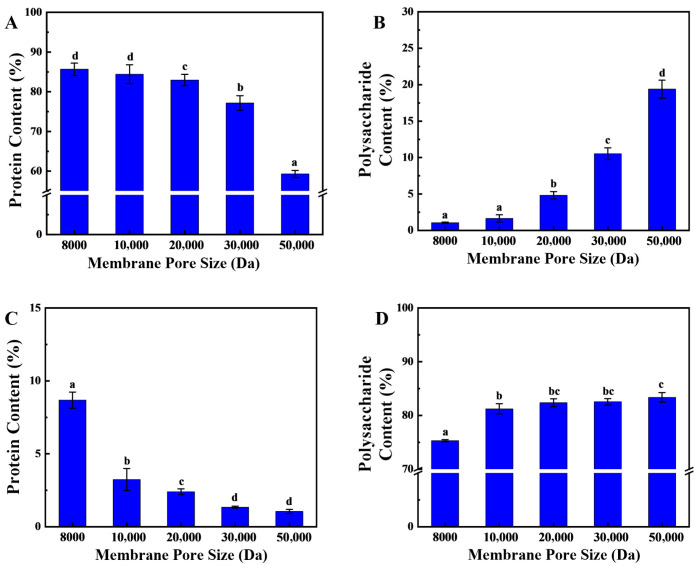
Separation of Sea Cucumber Polysaccharides and Peptides. (**A**,**B**) show the protein and polysaccharide contents in the retained fraction; (**C**,**D**) show the protein and polysaccharide contents in the eluted fraction. (Note: Lowercase letters in the figure indicate significant differences (*p* < 0.05) in the molecular weight of enzymatic hydrolysates from different enzymes).

The proteins in sea cucumber body walls are primarily collagen, which undergoes significant molecular weight reduction after enzymatic hydrolysis. Sea cucumber polysaccharides also constitute a vital component of the body wall, primarily residing in the extracellular matrix. They account for 6% of dry weight and mainly comprise two types, glycosaminoglycans and fucoidans, with natural molecular weight ranges of 40,000–50,000 and 80,000–100,000, respectively [23]. The substantial molecular weight difference between these two components after enzymatic hydrolysis enables their simultaneous separation via membrane filtration. In the membrane separation processes, as the pore size of the ultrafiltration membrane increases, the peptide content in the effluent first stabilizes above 85% and then gradually decreases to 62% (Figure 2A). The primary rationale for this phenomenon pertains to the molecular characteristics of the peptides resulting from secondary enzymatic hydrolysis, which characteristically exhibit smaller molecular weights. In contrast, sea cucumber polysaccharides, which have not undergone enzymatic hydrolysis, possess larger molecular weights and are retained by the ultrafiltration membrane. The results (Figure 2B) indicate that when the ultrafiltration membrane’s pore size exceeds 20,000 Da, there is a significant increase in the polysaccharide content of the effluent. At this juncture, sea cucumber polysaccharides traverse the larger pores, engendering a substantial augmentation in polysaccharide content in the effluent, concomitant with a diminution in peptide proportion.

Furthermore, the findings indicate that as the pore size of the ultrafiltration membrane increases from 8000 Da to 10,000 Da, the protein content in the retained fraction rapidly decreases from 7.8% to less than 3% and continues to decrease as the pore size is further increased (Figure 2C). This phenomenon can be attributed to the reduction in molecular weight of proteins with molecular weights above 10,000 Da following two-stage enzymatic hydrolysis. In comparison to polysaccharides, proteins exhibit a comparatively lower molecular weight, which results in the observed outcome. However, even as the pore size continues to increase, there is always 1.0% of protein-like substances in the retained fraction. These may be ultra-high-molecular-weight proteins or glycoprotein complexes that do not degrade easily. It has been demonstrated that when the ultrafiltration membrane’s pore size exceeds 10,000 Da, the polysaccharide content in the retained solution remains stable, exhibiting a significant difference from the retained solution produced by an 8000 Da ultrafiltration membrane (Figure 2D). This finding suggests that the molecular weight of the protein in the retained fraction primarily ranges between 8000 and 10,000 Da.

In summary, an ultrafiltration membrane with a pore size of 10,000 Da was selected for the one-step separation of sea cucumber polysaccharides and peptides. After ultrafiltration, the effluent was sea cucumber peptides, with a protein content of 84.43 ± 0.96%, while the retained fraction was sea cucumber polysaccharides, with a polysaccharide content of 82.23 ± 1.26%. Previous studies have often used ion exchange resins to separate and purify sea cucumber polysaccharides, which results in high costs and reduced recovery rates of target active substances. In this study, following hydrolyzing sea cucumber body wall proteins, membrane separation technology was employed to simultaneously isolate polysaccharides and peptides. This approach reduced recovery costs while maintaining the efficiency of target product recovery.

### 2.3. Improvement of the Process for Removing Aromatic Amino Acids from HFO of Sea Cucumber

**Figure 3:**
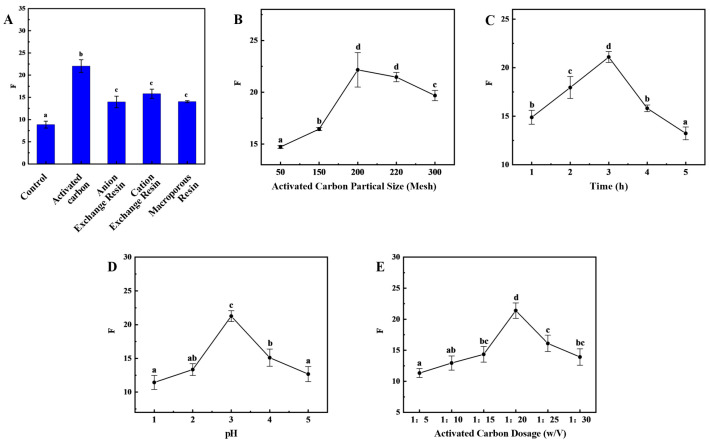
Improvement of the Process for Removing Aromatic Amino Acids from HFO of Sea Cucumber. F-value (short for F) is influenced by the type of adsorbent material (**A**), the mesh size of activated carbon (**B**), time (**C**), pH (**D**), and temperature (**E**). (Note: Lowercase letters in the figure indicate significant differences (*p* < 0.05) in the molecular weight of enzymatic hydrolysates from different enzymes).

Presently, the predominant approach for preparing HFO from protein hydrolysate mixtures involves the reduction in aromatic amino acids. This method is also applicable to sea cucumber collagen peptides. The results demonstrated that the adsorption efficiency exhibited variability among the various adsorbents (Figure 3A). The activated carbon demonstrated the most effective adsorption performance of the four adsorbents, exhibiting an OD220/OD280 ratio of 22.03 ± 1.43.

The impact of different grades of activated carbon on adsorption efficiency is significant. Among the tested grades, 200-mesh activated carbon demonstrated the optimal adsorption performance, exhibiting an OD220/OD280 ratio of 22.17 ± 1.66 (Figure 3B). During the initial stage of de-aromatization, as time progresses, there is an increase in the contact between the activated carbon surface and aromatic compounds. This, in turn, enhances the adsorption capacity of the activated carbon surface and significantly improves de-aromatization efficiency. As time progresses, the content of aromatic amino acids diminishes, concurrent with the onset of adsorption of branched-chain amino acids. This phenomenon results in a decrease in OD220/OD280. When the adsorption time reaches 3 h, OD220/OD280 reaches its maximum value of 21.09 ± 0.56 (Figure 3C). Conversely, an increase in pH results in an initial rise and subsequent decrease in the OD220/OD280 ratio (Figure 3D). At a pH of 3, the maximum OD220/OD280 ratio is 21.26 ± 0.80, a result primarily attributable to the surface characteristics of activated carbon, the nature of the adsorbate, and the chemical environment of the solution. The surface of activated carbon contains various functional groups, and the charge state of the activated carbon surface changes with pH. At lower pH values, the surface typically carries a positive charge, which may enhance the adsorption of negatively charged aromatic compounds. Conversely, at higher pH values, the surface carries a negative charge, which may enhance the adsorption of positively charged molecules. In the presence of an acidic environment, the adsorption of certain aromatic compounds, which are inherently difficult to ionize, may be enhanced. The pH of the medium exerts a discernible influence on the solubility of these compounds, with concomitant alterations in solubility potentially exerting a direct effect on the available adsorption capacity of the aromatic compounds. The incorporation of activated carbon has been demonstrated to augment the number of adsorption sites, thereby enhancing the capacity for the adsorption of aromatic compounds and the subsequent de-aromatization process. However, it should be noted that there is an optimal addition amount. Exceeding this value may result in a gradual decrease in de-aromatization efficiency or even reduced adsorption efficiency. This can be attributed to factors such as mutual interference between activated carbon particles and uneven distribution. Furthermore, the presence of excessive activated carbon has been shown to result in increased treatment costs and the potential for processing complications. The OD220/OD280 ratio demonstrates a substantial response to varying levels of activated carbon addition, exhibiting an initial increase followed by a subsequent decrease with increasing addition levels. At an activated carbon addition ratio of 1:20, the OD220/OD280 ratio reaches its maximum value of 21.40 ± 1.23 (Figure 3E).

A subsequent analysis of the amino acid composition both before and after activated carbon adsorption revealed a significant reduction in aromatic amino acids (Supplementary Table S1). This finding suggests that activated carbon is highly effective in removing aromatic groups. Subsequent to activated carbon adsorption, the F-value of the amino acid composition exhibited a substantial increase, with the F-value attaining 23.82 after adsorption, thereby satisfying the criteria for HFO (F-value > 20). Branched-chain amino acids (BCAAs) are a class of amino acids that have been identified as being particularly important for athletes and fitness enthusiasts. Among these BCAAs, leucine, isoleucine, and valine have been found to promote muscle synthesis, aid in muscle repair and growth, and are of particular importance in these contexts. Furthermore, BCAAs have been demonstrated to reduce fatigue during exercise, enhance endurance, and improve athletic performance. These substances have also been demonstrated to alleviate muscle soreness and accelerate recovery. The yield of HFO from sea cucumbers was measured to be 24.56% through validated testing. The amino acid residue sequence analysis results for HFO are shown in Supplementary Table S2.

### 2.4. HFO from Sea Cucumbers Enhance Motor Function in Mice

After statistical comparison of body weight and organ indices among the blank group (equivalent dose of distilled water), control group (small-molecule peptides from sea cucumber), and three experimental groups (low, medium, and high-dose HFO groups), no significant differences in mouse body weight were observed (Supplementary Table S2). This indicates that the substance does not affect mouse growth and development. No substantial disparities were observed in organ indices (Supplemental Table S3), thereby substantiating the safety of HFO for organ health and affirming their non-impact on mouse growth and development.

**Figure 4:**
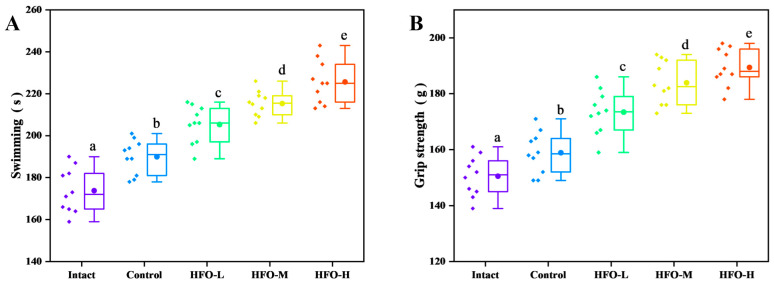
Effect of HFO on Exercise Performance of Mice. (**A**) Exhaustion Swimming Time, (**B**) Grip Strength. Three experimental groups—low, medium, and high-dose HFO groups—are abbreviated as HFO-L, HFO-M, and HFO-H, respectively. (Note: Lowercase letters in the figure indicate significant differences (*p* < 0.05) in the molecular weight of enzymatic hydrolysates from different enzymes).

Exercise endurance is a critical indicator of an organism’s capacity to resist fatigue. The mouse exhaustion swimming test, when utilized as a model for evaluating anti-fatigue effects, has been demonstrated to effectively reflect the exercise endurance level of mice. This test is frequently utilized to evaluate the anti-fatigue properties of compounds. The duration of swimming time exhibited a direct correlation with the compound’s anti-fatigue effect, suggesting a relationship between compound dosage and the extent of its ergogenic benefits [24]. The findings of the mouse weighted swimming test (Figure 4A) demonstrate that HFO can remarkably augment the exercise endurance of mice, with the swimming duration exhibiting a dose-dependent relationship.

The phenomenon of muscle loss can result in a state of fatigue during physical exercise, thereby reducing the duration of exercise. Nutritional intervention has been demonstrated to reduce muscle damage, thereby enhancing muscle strength and function, and alleviating fatigue [25]. The results of the mouse grip strength experiment (Figure 4B) demonstrate that HFO have a significant effect on enhancing the grip strength of the mouse’s forelimbs, thereby improving muscle endurance.

### 2.5. The Effect of HFO from Sea Cucumbers on Mouse Tissue Morphology

**Figure 5:**
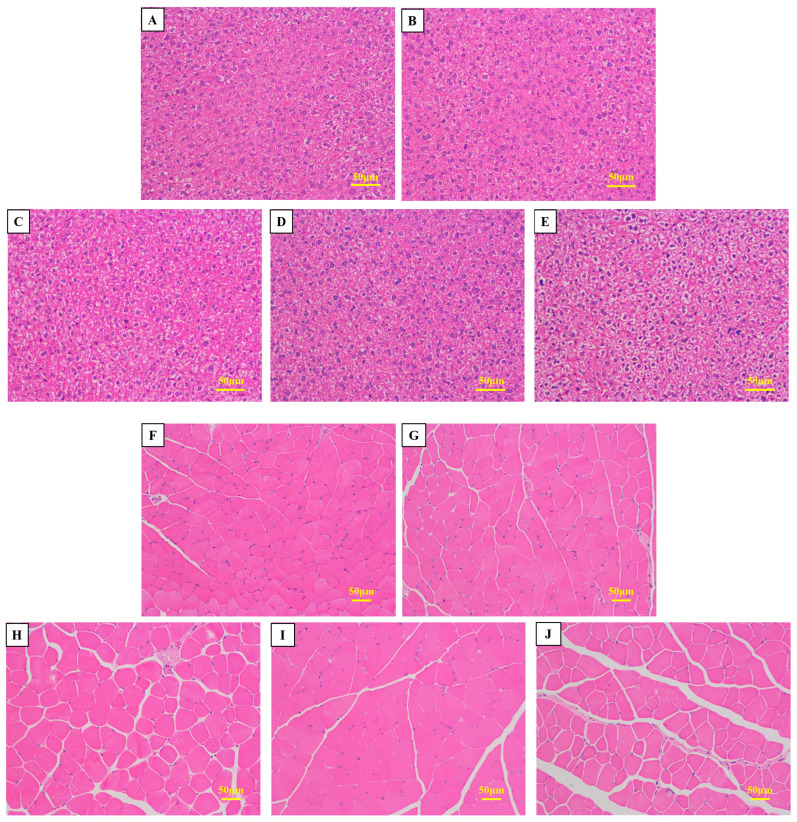
Morphological Observation of Mouse Liver Tissue and Muscle Tissue. (**A**,**F**) Blank Group; (**B**,**G**) Control Group; (**C**,**H**) Low-Dose Group; (**D**,**I**) Medium-Dose Group; (**E**,**J**) High-Dose Group.

In comparison with the control group, mice that were fed a diet containing sea cucumber small-molecule peptides and HFO exhibited normal liver tissue structure, with more compact hepatocyte organization and no obvious pathological changes (Figure 5A–E). These findings suggest that the administration of HFO does not induce pathological alterations in mouse livers and can also prevent liver damage caused by intense exercise. The administration of HFO derived from sea cucumbers to murine subjects resulted in a substantial augmentation in muscle fiber density within the gastrocnemius muscle. This observation was accompanied by a significant increase in the proportion of muscle fiber area within the gastrocnemius muscle (Figure 5F–J). This finding indicates that HFO have the potential to enhance muscle endurance and explosiveness in mice. A synthesis of the outcomes from prior grip strength and swimming experiments revealed substantial enhancements in forelimb grip strength and endurance among the test subjects. Histological analysis of the gastrocnemius muscle tissue revealed no obvious pathological changes, indicating that feeding HFO does not cause damage to the gastrocnemius muscle and has a significant protective effect, reducing damage to muscle tissue caused by intense exercise and markedly enhancing forelimb grip strength and exercise endurance in mice.

### 2.6. The Anti-Fatigue Mechanism of HFO in Sea Cucumbers

**Figure 6:**
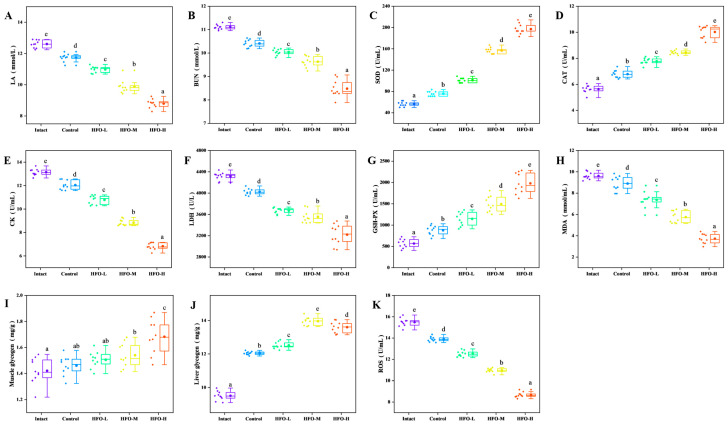
Effects of High F-value Oligopeptides from Sea Cucumber on Physiological Indices of Exercise-fatigued Mice. (**A**) Lactic Acid (LA) Levels; (**B**) Blood Urea Nitrogen (BUN) Levels; (**C**) Superoxide Dismutase (SOD) Activity; (**D**) Catalase (CAT) Activity; (**E**) Creatine Kinase (CK) Activity; (**F**) Lactate Dehydrogenase (LDH) Activity; (**G**) Glutathione Peroxidase (GSH-Px) Activity; (**H**) Malondialdehyde (MDA) Content; (**I**) Muscle Glycogen Content; (**J**) Liver Glycogen Content; (**K**) Reactive Oxygen Species (ROS) levels. (Note: Lowercase letters in the figure indicate significant differences (*p* < 0.05) in the molecular weight of enzymatic hydrolysates from different enzymes).

Intense physical exertion results in a substantial increase in muscle demand for oxygen. However, as the body’s oxygen supply is incapable of meeting the demands of muscle consumption, it is compelled to activate anaerobic metabolic pathways. This results in a swift escalation in glycolysis rates and the subsequent accumulation of substantial quantities of lactic acid [26]. The generation of substantial quantities of lactic acid results in a decrease in the pH level of blood within muscle tissue. This acidic environment has been demonstrated to exert a deleterious effect on cardiac circulatory function and skeletal muscle contraction capacity. Moreover, it has been identified as a pivotal factor contributing to exercise-induced fatigue [27]. As demonstrated in the results (Figure 6A), following exhaustive swimming, the lactic acid levels in the HFO group were significantly lower than those in the control group. The reduction in lactic acid levels exhibited a dose-dependent relationship. Furthermore, during periods of strenuous physical activity, if the body experiences a deficiency in glucose supplementation for an extended duration, it will begin to break down proteins to maintain essential bodily functions. Urea, the final product of protein metabolism, accumulates in significant quantities within the body during prolonged endurance exercise. Given the established negative correlation between urea nitrogen levels and exercise endurance, it can be concluded that an improvement in exercise tolerance is associated with reduced urea nitrogen levels. Consequently, the reduction in urea nitrogen levels in the body is of paramount importance for enhancing fatigue resistance [28]. The findings indicated a decline in urea nitrogen levels across all three groups (HFO low, medium, and high doses), with reductions of 9.63%, 13.32%, and 23.67%, respectively (Figure 6B). The high-dose group demonstrated optimal outcomes, exhibiting a discernible dose-dependent effect.

Creatine kinase, an essential enzyme in energy metabolism, plays a pivotal role in the body’s energy management. This enzyme catalyzes the process of phosphorylation of creatine, resulting in the formation of high-energy phosphocreatine. A notable function of phosphocreatine is the transfer of a phosphate group to adenosine diphosphate (ADP), resulting in the formation of adenosine triphosphate (ATP). This process is crucial for sustaining various bodily functions. During periods of physical exertion, the body experiences a significant demand for ATP, leading to its rapid consumption. In order to replenish sufficient ATP and sustain prolonged physical activity, the activity of creatine kinase—the enzyme regulating ATP production—increases, thereby generating more ATP and maintaining bodily functions, as well as delaying fatigue [29]. During periods of strenuous physical activity, muscle cells are subject to damage, which results in an elevated level of permeability within the cell membrane. Consequently, creatine kinase from muscle cells can be released into the bloodstream, resulting in an increase in serum creatine kinase activity. Serum creatine kinase, a product of muscle cells, is a crucial indicator of muscle cell damage, with higher levels directly reflecting the extent of injury [30]. As demonstrated in Figure 6E, the serum creatine kinase activities in the HFO low-, medium-, and high-dose groups were lower than that in the blank group and the control group (sea cucumber small-molecule peptides). In comparison with the blank group, serum creatine kinase activity in the HFO low-, medium-, and high-dose groups exhibited decreases of 17.94%, 32.77%, and 48.14%, respectively. This phenomenon also demonstrates a certain degree of dose-dependent relationship. The findings suggest that HFO derived from sea cucumbers can effectively protect muscle cells and prevent the leakage of creatine kinase from muscle cells into the bloodstream. Furthermore, HFO have been shown to augment ATP levels in the body while concurrently protecting muscle cells. This protective effect contributes to the attainment of an anti-fatigue effect.

Lactate dehydrogenase (LDH) is a pivotal enzyme in the reversible conversion of lactate to pyruvate, a process that occurs during anaerobic glycolysis and gluconeogenesis. It has been demonstrated that, particularly during periods of strenuous physical exertion, the activity of this enzyme is paramount for the efficient metabolism of lactate [31]. The tricarboxylic acid cycle (TCA) is a series of reactions in which pyruvate is converted into carbon dioxide (CO_2_) and water (H_2_O). These byproducts are subsequently excreted from the body, thereby reducing the burden on the body caused by excessive lactate accumulation. During periods of strenuous physical activity, the permeability of muscle cell membranes increases, resulting in the leakage of lactate dehydrogenase into the bloodstream. This, in turn, leads to an increase in the activity of lactate dehydrogenase in the serum. This phenomenon is attributed to the reduction in energy derived from lactate metabolism within muscle cells, resulting in excessive lactate accumulation and, consequently, fatigue in the body [32]. As illustrated in Figure 6F, in comparison with the blank group and the control group administered low-dose sea cucumber oligopeptides, the serum lactate dehydrogenase activity in the high-dose sea cucumber oligopeptide group was notably diminished, manifesting a dose-dependent relationship. In comparison with the blank group, the HFO low-, medium-, and high-dose groups exhibited reductions of 14.94%, 17.67%, and 25.43%, respectively. These reductions were found to be statistically significant and exhibited a certain degree of dose dependency. The findings suggest that HFO can effectively protect muscle cells and mitigate the damage caused by intense exercise.

Glycogen is considered to be one of the primary forms of energy storage in the body. Glycogen provides the body with energy through five mechanisms [33]: Firstly, during periods of intense physical exertion, a decline in blood glucose levels prompts the initiation of the hepatic glycogenolysis pathway. The process of glycolysis, initiated by the action of specific enzymes, involves the breakdown of liver glycogen into glucose, which is then released into the bloodstream. This process is crucial for maintaining blood glucose homeostasis and meeting the energy demands of cells during periods of physical exertion. During strenuous exercise or when oxygen supply is limited, muscle tissue can undergo a process of direct oxidation of glycogen for energy production through anaerobic respiration. This process, however, results in the production of lactic acid. The glycolytic pathway, which is predominantly located in the cytoplasm, plays a pivotal role in supporting various bodily functions. In the process of enzymatic catalysis, the transformation of glucose-1-phosphate into glucose-6-phosphate is initiated, thereby facilitating the entry of the glycolytic pathway and the subsequent production of a substantial amount of ATP. This process supplies energy to cells. The citric acid cycle, a series of biochemical reactions, occurs within cellular mitochondria, generating ATP as a byproduct. The pentose phosphate pathway, which plays a pivotal role in various metabolic reactions in vital organs such as the liver, breasts, and red blood cells, is another significant pathway. The significance of glycogen content in endurance exercise has been substantiated, underscoring the notion that the depletion of muscle glycogen plays a pivotal role in the onset of fatigue. Consequently, the glycogen content of a subject can serve as a significant indicator of their fatigue resistance. It has been demonstrated that an elevated level of glycogen within the body corresponds to an augmented degree of tolerance to physical exertion. As demonstrated in Figure 6I, the muscle glycogen content in the HFO low-, medium-, and high-dose groups exceeded that of the control group, thereby indicating that HFO promote glycogen accumulation in the body, with the high-dose group exhibiting the optimal effect. In comparison with the control group, the muscle glycogen content in the HFO low-, medium-, and high-dose groups exhibited increases of 5.63%, 8.45%, and 18.31%, respectively, in relation to the control group. These increases were found to be statistically significant. The medium-dose and high-dose groups exhibited significant differences in comparison with the control group. As illustrated in Figure 6J, the muscle glycogen content in the low, medium, and high-dose groups of HFO was higher than that in the control group. In comparison with the control group, the liver glycogen content in the low, medium, and high-dose groups of HFO increased by 31.57%, 46.94%, and 43.15%, respectively, as compared with the control group. The medium-dose group exhibited the most favorable outcomes. A comparison of the three HFO dose groups (low, medium, and high) with the control group revealed significant differences. In summary, the provision of oligopeptides with a high F-value to sea cucumbers has been demonstrated to elicit a marked increase in muscle and liver glycogen levels in murine subjects. This phenomenon is accompanied by an enhancement in energy metabolism, culminating in an augmented exercise tolerance capacity of the organism.

Superoxide dismutase (SOD) is a significant antioxidant enzyme that is present in living organisms. It has the capacity to eliminate free radicals. SOD catalyzes the conversion of superoxide anion radicals into H_2_O_2_ and O_2_, thereby protecting cells from oxidative damage [34]. Catalase, a component of the body’s natural defense system, has been shown to effectively eliminate reactive oxygen species, thus preventing oxidative damage induced by physical exertion. Catalase enzymes, present in the body, facilitate the decomposition of H_2_O_2_ into H_2_O and O_2_, thereby reducing the accumulation of hydrogen peroxide within cells and preventing damage to cellular structure and function [35]. Glutathione peroxidase, an important antioxidant enzyme in the body, plays a crucial role in maintaining redox homeostasis. Glutathione peroxidase catalyzes the reaction between reduced glutathione and peroxides, thereby eliminating peroxides in the body. This process helps alleviate oxidative stress-induced damage to cells and protects cellular health [36]. Consequently, the augmentation of antioxidant enzyme activity contributes to the mitigation of damage incurred by exercise-induced oxidative stress reactions to cells, thereby facilitating the alleviation of fatigue [37]. As shown in Figure 6C, the SOD activity in the HFO low, medium, and high-dose groups exhibited a significant increase compared to the blank group and the sea cucumber small-molecule peptide group. Additionally, a dose-dependent relationship was observed within a specific range. In comparison with the blank group, the SOD activity in the HFO low, medium, and high-dose groups exhibited increases of 1.79, 2.77, and 3.49 times, respectively, with significant differences, as demonstrated in Figure 6D, the three groups of HFO at low, medium, and high doses exhibited increased catalase activity compared to the blank group and sea cucumber small-molecule peptides. This increase in catalase activity showed a dose-dependent trend within a certain range. In comparison with the blank group, the catalase activity in the three groups of HFO at low, medium, and high doses increased by 38.01%, 49.91%, and 77.97%, respectively, indicating significant differences. As demonstrated in Figure 6G, the three groups of HFO at low, medium, and high doses exhibited significantly elevated glutathione peroxidase activity in comparison to the blank group and the sea cucumber small-molecule peptide group. This activity demonstrated a dose-dependent trend within a specific range. In comparison with the blank group, the glutathione peroxidase activity in the HFO low, medium, and high-dose groups increased by 2.01-, 2.67-, and 3.52-fold, respectively, with significant differences.

As indicated by research findings and experimental results, periods of high-intensity physical exertion have been demonstrated to result in substantial fluctuations in the levels of reactive oxygen species (ROS) within the human body. The increase in ROS has been demonstrated to exacerbate lipid peroxidation in liver and muscle tissues. Malondialdehyde (MDA) is a primary metabolic byproduct of lipid peroxidation in cell membranes. Consequently, the levels of MDA can serve as a reflection of the body’s antioxidant stress response, and alterations in MDA levels can also be utilized for the assessment of the extent of fatigue [38]. As demonstrated in Figure 6H, the content of MDA in the three groups that were administered HFO at low, medium, and high doses exhibited a significant decrease, with the most pronounced effect observed in the high-dose group. In comparison with the blank group, the MDA content in the three groups that were administered HFO at low, medium, and high doses exhibited a decrease of 1.29-, 1.67-, and 2.59-fold, respectively, in relation to the control group. These results were found to be statistically significant. As demonstrated by the preceding analysis, HFO derived from sea cucumbers have the capacity to augment the activity of antioxidant enzymes within the body, thereby leading to a reduction in reactive oxygen species. In addition, these peptides have been observed to inhibit lipid peroxidation in cell membranes, resulting in an anti-fatigue effect.

Some studies have demonstrated that reactive oxygen species (ROS), which include superoxide radicals, hydrogen peroxide, and their downstream byproducts such as peroxides and carboxylates, can induce protein oxidation in response to exercise, thereby contributing to muscle fatigue. Elevated levels of ROS within cells are also indicative of apoptosis. Under typical conditions, ROS are eliminated by the antioxidant system and maintained within a normal range. When there is an imbalance between oxidative and antioxidant processes, the body is unable to maintain a normal range of ROS, which can lead to oxidative stress. This, in turn, can result in damage to various macromolecules within the body [37]. As demonstrated in Figure 6, the levels of ROS in mice fed HFO at low, medium, and high doses exhibited a downward trend. This finding was concomitant with an increase in the activities of SOD, catalase, and glutathione peroxidase. The high-dose group fed HFO demonstrated the most optimal results, with a 43.99% reduction in reactive oxygen species in the body.

## 3. Materials and Methods

### 3.1. Experimental Materials

Fresh sea cucumbers (*Stichopus japonicus*) were provided by Beijing Tong Ren Tang Health (Dalian) Marine Food Co., Ltd. Fresh sea cucumbers from the Yellow Sea and Bohai Sea were immediately pretreated after being caught, with their internal organs and holothurian mouth removed, and then frozen under −20 °C for subsequent experiments.

### 3.2. Screening of Enzymatic Hydrolysis Conditions

The enzyme used in the first step of enzymatic hydrolysis process is selected from the following four enzymes based on the degree of hydrolysis results: neutral protease (from *Bacillus subtilis* strain 1393, 250,000 U/g) (pH = 7.0, 50 °C), pepsin (from pig stomach, 100,000 U/g) (pH = 3.0, 37 °C), trypsin (from pig pancreas, 100,000 U/g) (pH = 8.0, 37 °C), and alkaline protease (from *Bacillus licheniformis* strain 2709, 250,000 U/g) (pH = 9.0, 50 °C). The feed-to-enzyme ratio was 1:5, with an enzyme dosage of 5000 U/kg, and hydrolysis was conducted for a period of four hours. The degree of hydrolysis of the four proteases was compared [39].

Subsequently, using the protease selected in the previous step, its enzymatic hydrolysis conditions were optimized, including hydrolysis time (1 h, 2 h, 3 h, 4 h, 5 h), feed-to-liquid ratio (1:3, 1:4, 1:5, 1:6, 1:7), enzyme dosage (2000 U/kg, 3000 U/kg, 4000 U/kg, 5000 U/kg, 6000 U/kg), pH value (6.0, 6.5, 7.0, 7.5, 8.0) and the range of temperatures (40 °C, 45 °C, 50 °C, 55 °C, 60 °C, 65 °C).

In the second step of the enzymatic hydrolysis process, flavourzyme (from *Aspergillus oryzae*, 200,000 U/g) (pH = 7.0, 55 °C), bromelain (from pineapple, 200,000 U/g) (pH = 7.0, 55 °C), and papain (from papaya, 200,000 U/g) (pH = 7.0, 60 °C) were utilized. In the context of a solid-to-liquid ratio of 1:5 and an enzyme dosage of 5000 U/kg, the process of enzymatic hydrolysis was conducted for a duration of four hours. Based on the degree of hydrolysis results of the three enzymes, the optimal second-stage hydrolysis enzyme was selected.

Based on this, the enzymatic hydrolysis conditions for the second stage were optimized, including hydrolysis time (1 h, 2 h, 3 h, 4 h, 5 h), solid-to-liquid ratio (1:3, 1:4, 1:5, 1:6, 1:7), enzyme dosage (2000 U/kg, 3000 U/kg, 4000 U/kg, 5000 U/kg, 6000 U/kg), pH value (6.0, 6.5, 7.0, 7.5, 8.0), and the temperature (45 °C, 50 °C, 55 °C, 60 °C, 65 °C).

All kinds of proteases were purchased from DOING-HIGHER Biotechnology Co., Ltd. (Nanning, China).

### 3.3. Determination of Degree of Hydrolysis

The degree of hydrolysis (DH) has been shown to be an indicator of the progress of the hydrolysis reaction. The TNBS method is utilized to ascertain the extent of hydrolysis, with minor adjustments [40,41]. A quantity of 0.2 g of fresh sea cucumber should be added to 10 mL of hydrochloric acid (6 mol/L), and then which were hydrolyzed at 120 °C for 24 h as fully hydrolyzed samples. After that the pH was adjusted to 7. The fully hydrolyzed samples were then diluted 50-fold. In order to conduct the experiment, 50 μL of the fully hydrolyzed sample and each time point sample must be taken. Thereafter, 0.5 mL of phosphate buffer (pH = 8.0) and 0.5 mL of 0.05% TNBS must be added. The samples should then be reacted in the dark at 50 °C for 1 h. Subsequent to the occurrence of the reaction, the addition of 1 mL of 0.1 mol/L HCl is to be affected without delay in order to effect termination of the reaction. The measurement of the absorbance at 420 nm is then to be undertaken. DH was calculated according to the following formula:

$$\mathrm{DH}\left(\%\right)=\frac{{OD}_{s}\;-{\;OD}_{b}}{{OD}_{t}\;-\;{OD}_{b}}\times 100\%$$

where DH means the degree of hydrolysis for the sample, %;

*OD_s_* means the absorbance of the sample;

*OD_t_* means the absorbance of fully hydrolyzed sample;

*OD_b_* means the absorbance of blank.

### 3.4. Determination of Molecular Weight Distribution

The determination was made using gel permeation chromatography [42]. The enzymatic products were dissolved in ultrapure water at a concentration of 2 mg/mL, then filtered through a 0.22 μm pore size membrane to remove insoluble impurities. Molecular weight measurement was performed using high-performance liquid chromatography (HPLC) equipped with a TSK-gel G2000-SWxl column (TOSOH, Tokyo, Japan) (7.8 × 300 mm). A 10 μL sample was injected into the injector, and the ultraviolet (UV) detector was set to 214 nm. The mobile phase was constituted of a 45% acetonitrile aqueous solution containing 0.1% trifluoroacetic acid, with a flow rate of 0.5 mL/min. L-lysine (146 Da), vitamin E (468 Da), octapeptide (1078 Da), peptidase inhibitor (6511 Da), and cytochrome C (12,384 Da) were selected as standards. A standard curve was plotted by log-fitting the retention times and molecular weights of the standard samples. The molecular weights of the sea cucumber enzymatic hydrolysis products were calculated. The standard curve equation was obtained by log-fitting the retention times and molecular weights of the standard samples, yielding Y = −0.2283X + 6.906. It is evident from the data that R^2^ takes on a value of 0.9994.

### 3.5. Simultaneous Separation of Sea Cucumber Polysaccharides and Peptides

It is evident that the sea cucumber enzymatic hydrolysate principally employs proteases for the purpose of enzymatic hydrolysis. Consequently, the sea cucumber polysaccharides remain intact and are not hydrolyzed. In view of the marked disparity in molecular weight between sea cucumber polysaccharides and peptides, membrane separation technology was selected for the simultaneous separation of the aforementioned polysaccharides and peptides. An ultrafiltration plate pilot-scale device (SUS316, Xiamen Fumei Technology Co., Ltd., Xiamen, China) was employed, and PES membranes with pore sizes of 8000 Da, 10,000 Da, 20,000 Da, 30,000 Da, and 50,000 Da (Pall Filter (Beijing) Co. Ltd., Beijing, China) were used to compare the effects of membrane pore size on the purity of polysaccharides and peptides after simultaneous separation.

### 3.6. Improvement of the De-Aromatization Process for HFO

As branched-chain amino acids have a maximum characteristic absorption peak at 220 nm and aromatic amino acids at 280 nm, the de-aromatization effect was determined by measuring the absorbance values at 220 nm and 280 nm of the de-aromatized samples using a UV spectrophotometer and calculating the ratio of the two values [43].

Activated carbon (pH = 3.0, 30 °C), anion exchange resin (pH = 7.0, 30 °C), cation exchange resin (pH = 7.0, 30 °C), and macroporous adsorption resin (pH = 7.0, 30 °C) were utilized in adsorption experiments with a feed-to-bed ratio of 1:20 and an adsorption time of 3 h. The following experiment was conducted: the absorbances of the de-aromatized samples at 220 nm and 280 nm were measured, in order to compare the effects of different adsorbents on the F value.

Subsequent experiments were conducted using activated carbon grades (50 mesh, 150 mesh, 200 mesh, 220 mesh, 320 mesh), adsorption time (1 h, 2 h, 3 h, 4 h, 5 h), adsorption pH (1, 2, 3, 4, 5), and adsorption feed-to-bed ratio (1:5, 1:10, 1:15, 1:20, 1:25, 1:30) to optimize the adsorption de-aromatization process.

Activated carbon (50 mesh, 150 mesh, 200 mesh, 220 mesh, 320 mesh), anion exchange resin (i-Quip™ D50), cation exchange resin (i-Quip™ CM40), and macroporous adsorption resin (Amberlyst^®^ A21) were all purchased from Aladdin Biochemical Technology Co., Ltd., Shanghai, China.

### 3.7. Animal Experiment Design

All animal experiments were conducted in strict accordance with the experimental protocol that had been approved by the Animal Ethics Committee of Dalian University of Technology (Approval Number: DLPU2024112). The experimental process complied with national standards for animal ethics. Sixty SPF-grade wild-type C57BL/6J mice (6–8 weeks old, 20 ± 2 g) were procured from Liaoning Changsheng Biotechnology Co., Ltd. Mouse feed and bedding were also procured from Liaoning Changsheng Biotechnology Co., Ltd., Benxi, China Location of animal housing: Dalian University of Technology. The environmental parameters for the housing of animals are as follows: a temperature of 22 ± 2 °C and a relative humidity of 55 ± 5% RH. Subsequent to the day of purchase, the mice were acclimatized for a period of seven days. Following acclimatization, the subjects were randomly divided into five groups, with 12 mice per group. The groups were set as follows: Blank group (Intact, administered an equal dose of distilled water via gavage); Control group (Control, administered sea cucumber oligopeptides via gavage, 50 mg/kg); High F-value oligopeptide low-dose group (HFO-L, administered high F-value oligopeptides via gavage, 50 mg/kg); High F-value oligopeptide medium-dose group (HFO-M, gavage with high F-value oligopeptides, 100 mg/kg); and High F-value oligopeptide high-dose group (HFO-H, gavage with high F-value oligopeptides, 200 mg/kg).

### 3.8. Mouse Body Weight and Organ Indices

Subsequent to the commencement of the experiment, the mice were weighed and recorded on a weekly basis. At the conclusion of the experiment, six mice from each group were subjected to a thorough dissection, during which the heart, liver, spleen, lungs, and kidneys were meticulously extracted. Subsequently, the organs were then rinsed with physiological saline, dried, and weighed. The organ indices were calculated based on the body weight of each mouse [44].

### 3.9. Grip Strength Test and Exhaustion Swimming Test

On the 28th day, a forelimb grip strength test was conducted, with each mouse undergoing three trials using a grip strength meter. The resulting data was recorded for each mouse [45]. On the 29th day, the swimming test was conducted to measure the duration of weight-bearing swimming after gavage [46]. A weight equivalent to 5% of the mouse’s body weight was attached to the tail, and the mouse was placed in a pool with a water depth of at least 30 cm, maintained at 25 ± 2 °C. The time taken for each mouse to reach exhaustion during the swimming test was meticulously recorded.

### 3.10. Biochemical Analysis

Following the exhaustive swimming test and a 10 min rest period, mice were euthanized by cervical dislocation. Blood samples were immediately collected via eyeball vein puncture. The obtained blood was allowed to stand at room temperature for 4 h, then centrifuged at 4400× *g* for 15 min at 4 °C. The supernatant was aspirated and stored at −80 °C for biochemical analysis. Following blood collection, the mouse liver and gastrocnemius muscle were harvested and rinsed thoroughly with physiological saline, and a minimal portion excised for histological sectioning. The remaining tissue was homogenized in a homogenizer with an appropriate volume of homogenization buffer. The homogenate was centrifuged at 4 °C and 11,000× *g* for 20 min. The supernatant was aspirated and stored at −80 °C for biochemical analysis. The following parameters were measured in the study: lactic acid, blood urea nitrogen (BUN), liver glycogen and muscle glycogen, reactive oxygen species (ROS), malondialdehyde (MDA) content, creatine kinase (CK), lactate dehydrogenase (LDH), superoxide dismutase (SOD), catalase (CAT), and glutathione peroxidase (GSH-Px) activity [47]. All measurements were made using commercial assay kits from the Nanjing Jiancheng Biotechnology Research Institute (Nanjing, Chian).

### 3.11. Histological Observation

The mouse liver and muscle tissues were extracted in Section 3.10, thoroughly washed with physiological saline, and immediately fixed in 10% formalin solution for a period of 24 h. Following a process of dehydration with graded ethanol, the tissues were embedded in paraffin. Subsequently, liver and muscle tissues were sectioned (4 μm thick) and stained. The images were captured using an optical microscope equipped with a camera function [48].

### 3.12. Statistical Analysis Methods

Data from chemical experiments, including degree of hydrolysis, protein content, polysaccharide content, and chromatographic analysis, were obtained from three samples per group. Data from mouse experiments were generated from six parallel determinations per group. All experimental data are presented as mean ± standard deviation (mean ± SD) and analyzed statistically using SPSS 19.0 software (SPSS Inc., Chicago, IL, USA). The generation of charts was facilitated by employing OriginPro 2021 software (Origin Lab Corporation, Northampton, MA, USA). One-way analysis of variance (ANOVA) was employed to ascertain any discrepancies between the experimental groups, with subsequent multiple comparison procedures conducted using Duncan’s multiple range test. When the *p*-value was less than 0.05 (*p* < 0.05), the difference between groups was considered to be statistically significant.

## 4. Conclusions

The improved two-step enzymatic hydrolysis conditions employed a dual-enzyme system comprising neutral protease and flavor protease for hydrolysis, achieving a hydrolysis degree of 54.6 ± 1.55%. Membrane separation technology was utilized to enable low-cost simultaneous separation of sea cucumber polysaccharides and peptides. The 10,000 Da molecular weight membrane exhibited optimal separation efficacy, yielding a peptide content of 84.43 ± 0.96% and a polysaccharide content of 81.23 ± 0.96%. The yield of sea cucumber peptides was 45 ± 0.12%. After undergoing de-aromatization treatment with activated carbon, a substantial decline in the content of aromatic amino acids was observed, with branched-chain amino acids measuring 7.745 ± 0.449 mg/mL and aromatic amino acids measuring 0.332 ± 0.008 mg/mL, resulting in an F value of 23.82, and the yield of high-F-value oligopeptide from sea cucumbers was found to be 24.56%. The investigation revealed that high-F-value oligopeptide exhibited a substantial enhancement in the endurance and explosiveness of mice during exercise. The elimination of metabolic by products generated by fatigue is a potential property of high-F-value oligopeptides. High-F-value oligopeptides were also demonstrated to enhance the activity of antioxidant enzymes and glycogen reserves within the body, thereby enhancing the body’s capacity to neutralize free radicals and extend the duration of exercise, thus exerting an anti-fatigue effect. In addition, it was observed that high-F-value oligopeptides prevented exercise-induced liver damage and exhibited a protective effect, reducing exercise-induced muscle damage in mice. The aforementioned results will provide substantial support for the development and utilization of marine biological resources, the research and development of health products, and further scientific research. Notably, this study demonstrates that high-F-value oligopeptides from sea cucumbers significantly alleviate fatigue-induced damage in mice through physiological indicators. However, further investigation is warranted to identify the specific key peptide segments and their receptor-mediated functional activities within various fatigue-relieving signaling pathways. Such structure–activity relationship studies hold significant implications for the development of third-generation nutritional health foods. Additionally, the polysaccharide components obtained through ultrafiltration may also possess important functional activities. Given this study’s focus on peptide functionalities, subsequent research should also delve into the functional activities of these polysaccharides.

## Data Availability

The original contributions presented in this study are included in the article/Supplementary Material. Further inquiries can be directed to the corresponding author.

## Supplementary Materials

The following supporting information can be downloaded at https://www.mdpi.com/article/10.3390/md24010010/s1. The following supporting information was shown in Figure S1: Standard curve for determining the molecular weight distribution of peptides by gel permeation chromatography, and the molecular weight distribution of peptides in enzymatic hydrolysates after neutral protease, flavor protease, and dual-enzyme hydrolysis; Table S1: Amino Acid Content of High F-Value Oligopeptides; Table S2: Effect of HFO on Body Weight of Mice. Table S3: Effect of HFO on Body Weight of Mice; Table S4: Effect of HFO on Organ Index of Mice.

## Abbreviations

The following abbreviations are used in this manuscript:

HFO	High F-value oligopeptides
BCAAs	Branched-chain amino acids
LDH	Lactate dehydrogenase
TCA	tricarboxylic acid cycle
SOD	Superoxide dismutase
ROS	reactive oxygen species
MDA	Malondialdehyde
