# Supplementary material for: Enzymatic Hydrolysis-Assisted Separation and Purification of High F-Value Oligopeptides from Sea Cucumbers and Their Anti-Fatigue Mechanism
Source: Mar Drugs. 2025 Dec 23;24(1):10. doi: 10.3390/md24010010 (PMC12842808; doi:10.3390/md24010010)
Supplement: Supplementary file 1 [file marinedrugs-24-00010-s001.zip › marinedrugs-3834064-supplementary.pdf]

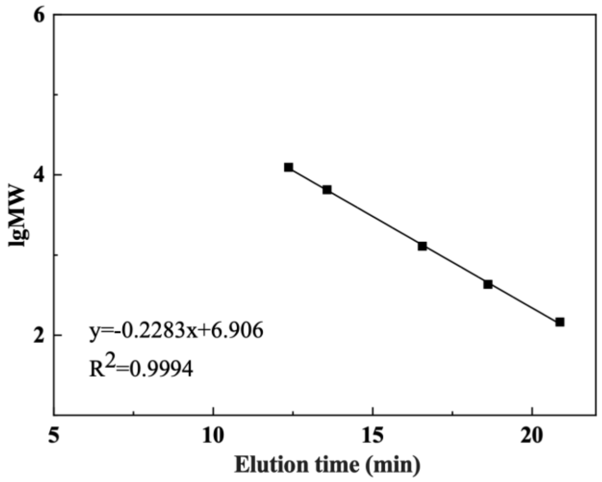

(A)

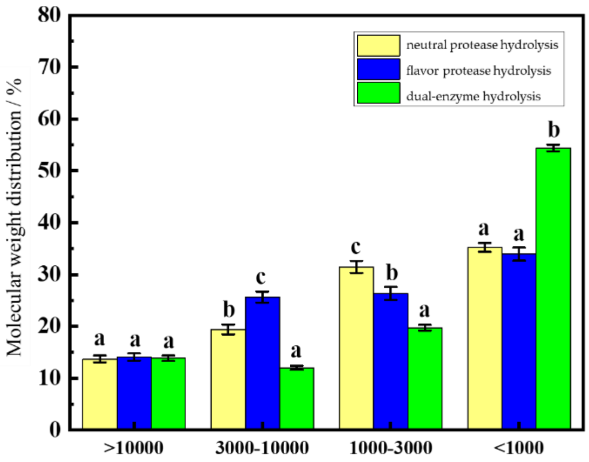

(B)

Supplementary Figure S1. (A) Standard curve for determining the molecular weight distribution of peptides by gel permeation chromatography, (B)The molecular weight distribution of peptides enzymatic hydrolysates after neutral protease, flavor protease, and dual-enzyme hydrolysis. (Lowercase letters in the figure indicate significant differences (p<0.05) in the molecular weight of enzymatic hydrolysates from different enzymes.)

Supplementary Table S1. Amino Acid Content of High F-Value Oligopeptides

**Amino Acid**	**Content (mg/mL)**	
	**Before adsorption**	**After adsorption**
	Essential amino acid	
Thr	3.633±0.151	2.450±0.229
Lys	1.945±0.218	1.760±0.096
Met	0.344±0.016	0.255±0.020
Phe○	1.209±0.079*	0.275±0.007*
Ile#	2.904±0.157	2.180±0.256
Leu#	2.953±0.074	2.913±0.085
His	0.711±0.025	0.578±0.040
Tyr○	2.498±0.115*	0.057±0.001*
Val#	2.783±0.136	2.652±0.108
Cys	0.455±0.036	0.273±0.052
	Non-essential amino acid	
Ser	1.760±0.020	1.100±0.131
Arg	1.208±0.201	1.073±0.079
Asp	3.425±0.055	2.012±0.147
Gly	1.572±0.024	1.346±0.289
Glu	4.008±0.052	3.442±0.222
Ala	4.113±0.457	2.768±0.168
Pro	1.906±0.277	1.241±0.064
Total (Essential + Non-essential)	37.426±2.093	26.375±1.993
	Branched-chain amino acids (BCAAs) and aromatic amino acids (AAAs)	
#BCAAs	8.640±0.367	7.745±0.449
○AAAs	3.707±0.194	0.332±0.008

Supplementary Table S2. Amino acid sequence of high F-value oligopeptides after activated carbon adsorption

No.	Sequence	No.	Sequence	No.	Sequence
1	TPEKNFGY	104	VPVILVGNK	206	DSLDLPH
2	DPPEQPGKP	105	IDLGDIKE	207	GLRL
3	NPPLIPKL	106	EELAKTQF	208	GIRL
4	TPPAPPDE	107	ADPVRTEP	209	GLRI
5	LPIGHLLSN	108	APTEIEK	210	TVPPAAVP
6	SGSSLHIGD	109	MDLNTRY	211	YPLPGDLKA
7	GPADVYKY	110	EFEAIKT	212	GLFEPSH
8	GTEVAVLGH	111	IPDWKP	213	VPLLEGKL
9	DDAPRAVFP	112	EDGEKVLE	214	ESLREP
10	APEEHPVL	113	DDFKGF	215	GEADKGLQF
11	PAPPPPPPP	114	LPIGHLLS	216	IPDEIRDS
12	VPVFINH	115	DHIPGSP	217	NRVV
13	GLDFTGVRN	116	NELRVAP	218	NDKDVPGSP
14	APSDKVSY	117	APSIIPLLQ	219	GQKGVLLP
15	DPLGLPGRP	118	VDLEPGTMD	220	DGYKW
16	EAGIKGIFQ	119	DDTRIVL	221	GQEQKYSQ
17	DVESLTKF	120	EEPPVVE	222	DTRF
18	SDEFVAAMD	121	GGDELPPTP	223	DELHLN
19	NPEALMPKS	122	ERELPVA	224	EAPLNPKAN
20	DLTSLKAEN	123	YGLVIKS	225	WPLYK
21	GDEGRPGP	124	EPPVVEV	226	TPNLERLA
22	DESGPSIVH	125	GVIHVID	227	EGKVIE
23	DSVIGNADY	126	DELMIEK	228	RDLTDY
24	DEGRPGP	127	SEFEAIK	229	VPLITK
25	DHTPGDPVP	128	DEAKIAL	230	QALEKLF
26	GPPVETPEP	129	LPVQLQR	231	EQYFKT
27	DDPSIPKT	130	DHDLVLDM	232	VPSVLK
28	LPTIPIHP	131	GYALPH	233	ELHL
29	DGQVITIGN	132	PVPPPPPP	234	AVGMLAGP
30	GSSLHIGD	133	PSIPKT	235	LVPR
31	DLISLVSKT	134	GDVNIKPL	236	PGKPEF

32	TEAPLNPKA	135	EQYFKT	237	ELFAALQ
33	ADIAEESLK	136	YDPNALR	238	EDGELRF
34	DMKVFSKP	137	YPPPLARF	239	DLTSLKA
35	ESLREPSTA	138	APGKGILAA	240	DGIRY
36	SPHVAGAAA	139	DPPRIDD	241	DGLRY
37	MPGERQY	140	RPSVNF	242	LPGNKYG
38	GDLERAYL	141	VPISGFHGD	243	ELFK
39	EPPNQSPTE	142	DKPDPSY	244	DLTSLKAE
40	APLNPKA	143	DGYKWIA	245	IPLK
41	FPEYPKN	144	ETLDGLAKT	246	LPLK
42	DEYGKIAG	145	TSCSPGGL	247	DVRY
43	ADSKIIPLN	146	IDLVEIKT	248	DDRAPSPTP
44	ADGGLIGHL	147	GVKAFP	249	DHDLVL
45	DDEFDIKT	148	DIPDEIRD	250	TPNDIVKG
46	SDPVAIQQ	149	TDYLMK	251	APPLKLN
47	SPVATLISK	150	VPFFMR	252	TPLHLAA
48	SEFEAIKT	151	APRAVF	253	GEDHIPGSP
49	APLNPKAN	152	VPSPKVSD	254	APLNPK
50	GERGPSGP	153	LPFEVRQ	255	GEAHNVFP
51	NPYTVKV	154	DGLRIDT	256	ILSAPR
52	DEGRPGPT	155	EIFIGGR	257	EKFPPP
53	TPDGKPVQP	156	QLEKLFE	258	LPIGH
54	DPWGVKV	157	NPIWLSPT	259	DLWR
55	STPEKNF	158	FPEYPK	260	GVHIPT
56	GVIPDWKP	159	APPERP	261	EFRQLN
57	GIHETTYN	160	QDFIELLN	262	DGPKL
58	ADQEIPGSP	161	VDKAMI	263	DGPKI
59	YDELHLN	162	DSINKEF	264	LEKLF
60	ESLREPST	163	DPSIPKT	265	DSLSILH
61	SPPEYFA	164	LPIGHLL	266	MDHDLVL
62	SYLIGTAKF	165	GPLTVLK	267	EVLK
63	DPVLIPKL	166	VPSPKVS	268	LPGNKY
64	EWSDRLP	167	KTLP	269	EDNLTFK
65	EDHIPGSP	168	YDEKFA	270	DSLRF
66	GDLPGKDAP	169	ADSLSILH	271	EPPIVDVNT

67	SPGLKIQP	170	IPNNVKT	272	QSCSAGLF
68	VPLPGFDK	171	DSLGSHSDP	273	APPTMKI
69	EHLLQGPE	172	QGIRGPSGP	274	DPKDPL
70	SEIDLGDIK	173	DEPEDPPP	275	ELIR
71	GSQDEPLKL	174	EQGPPGEPG	276	ELLR
72	TPEKNF	175	PPIICA	277	EILR
73	LPKMGQ	176	TPKNLF	278	TRLGLGF
74	VPNFHLP	177	AGNDFQLQP	279	APALF
75	APRAVFP	178	ERPYYGP	280	IPDKI
76	EGRPGPTGP	179	EYFK	281	EVDDEGDE
77	EELGITIGN	180	LPIGHL	282	LPGYKLPE
78	EQYFKTT	181	DYFHP	283	YDMKVF
79	DGPDKIP	182	DKDVPGSP	284	SPEIQEKL
80	PAPPPPPP	183	DPQGLPNRP	285	GGKFDP
81	VPNFH	184	MPRP	286	EKVLE
82	DEPPIVD	185	DLAALEK	287	QIDENLR
83	SSLHIGD	186	EPPPSKP	288	TVMCCAP
84	GPGAIIKLN	187	KTVI	289	VGSFSSPNY
85	APPTMK	188	KTVL	290	DDINLH
86	GDVNIKP	189	DSIRPTE	291	QETNINNL
87	EQYFKTT	190	SRLF	292	EIEYKP
88	NEPVKQF	191	SRIF	293	GPQGPPGP
89	YPLPGDLK	192	TVGPGSAAY	294	IPRIGS
90	DEKAIINV	193	VPFPRL	295	SPYVKL
91	DDPSIPK	194	DHDLVLD	296	EPQNPVSL
92	GTIPEQDP	195	PPVSF	297	ETRFE
93	APETVKL	196	ENPPVVT	298	LPLVKE
94	DDGIVRY	197	TKIP	299	DLRF
95	TPLGLVK	198	TPTIEGTFD	300	IPVIDK
96	DSLRFGAS	199	DVASTMGAM	301	GLVPLDK
97	GIKGIFQ	200	TPKNTP	302	QPPTVVP
98	QDPVERVP	201	KDFVVDP	303	PHAI
99	EFPEYPK	202	DPSIPK	304	ELHIGD
100	NPITYFG	203	DLDIYH	305	IPPPPPP
101	DHLIQLD	204	DEPFPPEE	306	GLFR

102	VPVTVVKE	205	GEDTKVV	307	GIFR
103	APHTWTF				

Supplementary Table S3. Effect of HFO on Body Weight of Mice

Groups	Body Weight (g)			
	**7d**	**14d**	**21d**	**28d**
Blank	21.18±0.37	22.51±0.43	23.49±0.36	25.52±0.42
Control	21.23±0.37*	22.35±0.50*	23.55±0.42*	25.45±0.45*
HFO-L	21.28±0.37*	22.46±0.48*	23.41±0.44*	25.44±0.42*
HFO-M	21.24±0.42*	22.39±0.42*	23.55±0.38*	25.60±0.41*
HFO-H	21.25±0.33*	22.41±0.40*	23.73±0.39*	25.23±0.56*

* No significant difference compared to the control group, $p<0.05$

Supplementary Table S4. Effect of HFO on Organ Index of Mice

Groups	Organ Index (mg/g)				
	Heart	Liver	Spleen	Lung	Kidney
Blank	5.33±0.83	43.40±3.12	4.72±0.75	35.30±1.47	14.66±0.88
Control	5.38±0.47*	43.73±2.91*	4.66±0.74*	35.26±1.16*	14.96±1.00*
HFO-L	5.30±0.54*	42.79±2.87*	4.27±1.25*	34.79±1.24*	14.81±0.86*
HFO-M	5.78±0.61*	41.20±2.13*	3.96±0.67*	35.41±1.19*	14.89±0.66*
HFO-H	5.52±0.49*	43.18±2.62*	4.44±0.70*	35.68±1.62*	14.71±0.94*

* No significant difference compared to the control group, $p<0.05$
